# Supplementary material for: Identification of type VI secretion system effector-immunity pairs using structural bioinformatics
Source: Mol Syst Biol. 2024 Apr 24;20(6):6. doi: 10.1038/s44320-024-00035-8 (PMC11148199; doi:10.1038/s44320-024-00035-8)
Supplement: Supplementary file 3 — Movie EV1 [file 44320_2024_35_MOESM3_ESM.zip › Movie EV1 Legend II.docx]

**Movie EV1. Time-lapse microscopy shows cells rounding and bursting.** Three scenes are shown, the first two are of the same visual field. The first only shows membrane staining (FM1-43), while the second shows membrane and nucleic acid stains (DAPI). The third shows membrane staining only.
