# Supplementary figures and images for: Identification of type VI secretion system effector-immunity pairs using structural bioinformatics
Source: Mol Syst Biol. 2024 Apr 24;20(6):6. doi: 10.1038/s44320-024-00035-8 (PMC11148199; doi:10.1038/s44320-024-00035-8)

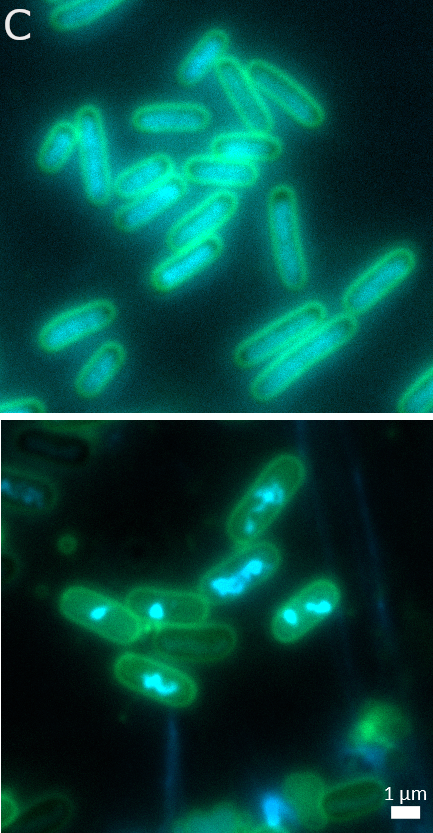

Supplement: Supplementary file 5 — Source data Fig. 4 [file 44320_2024_35_MOESM5_ESM.zip › MSB-2024-12200_SourceDataFor_Fig4C_labelled.tif]

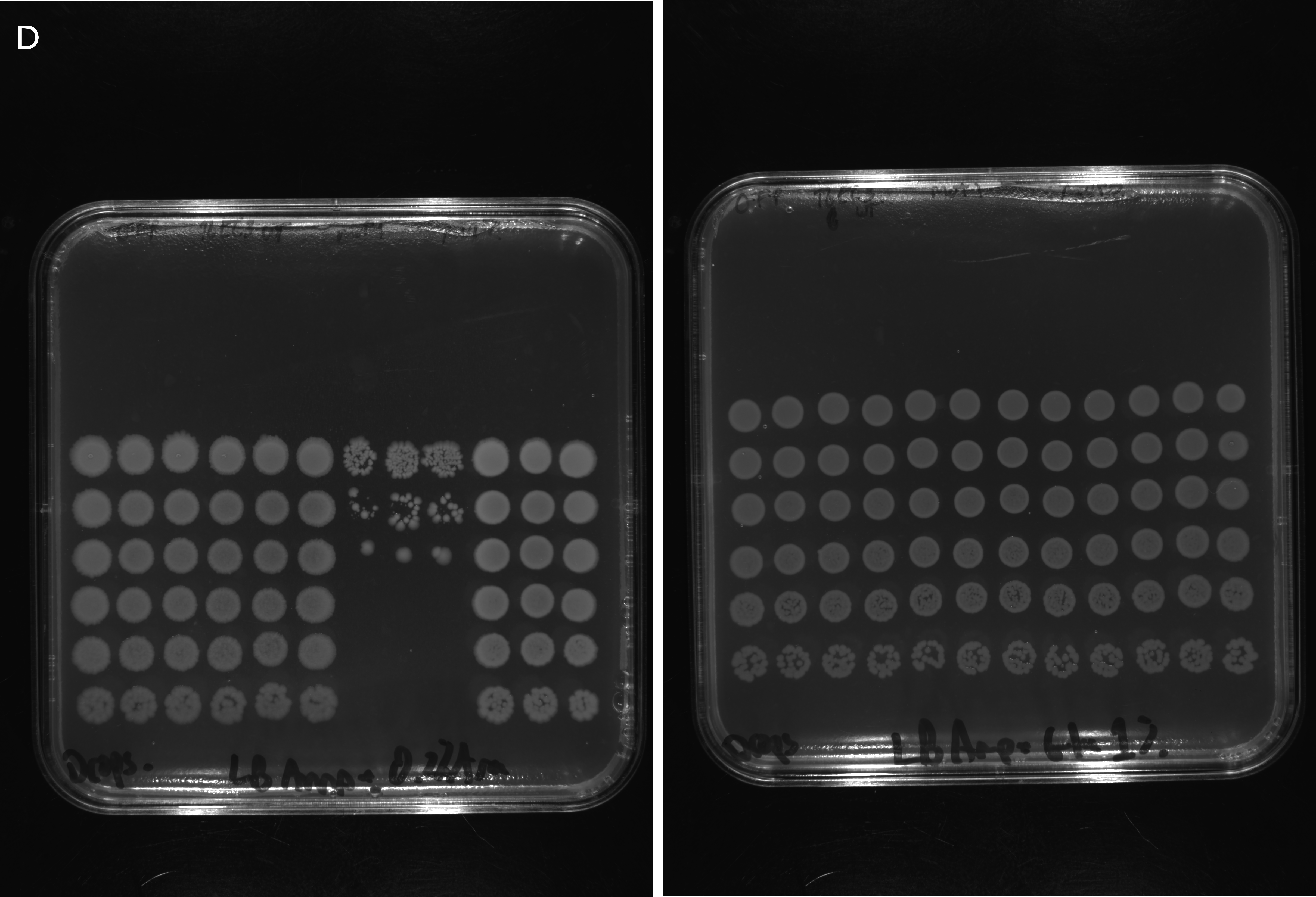

Supplement: Supplementary file 5 — Source data Fig. 4 [file 44320_2024_35_MOESM5_ESM.zip › MSB-2024-12200_SourceDataFor_Fig4D_labelled.tif]

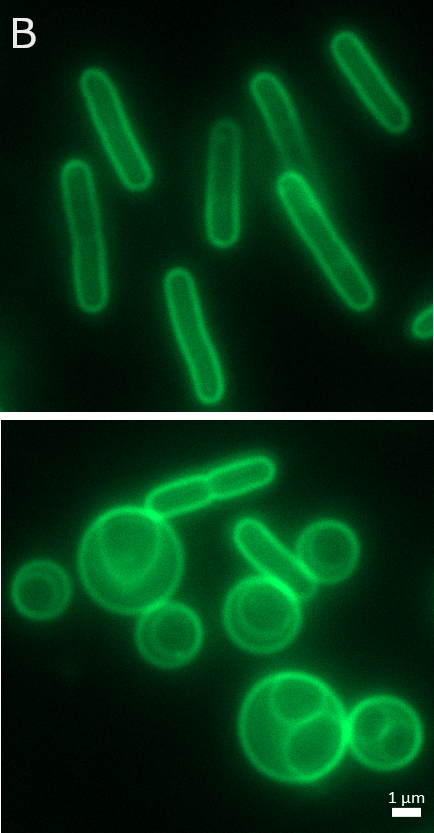

Supplement: Supplementary file 5 — Source data Fig. 4 [file 44320_2024_35_MOESM5_ESM.zip › MSB-2024-12200_SourceDataFor_Fig4B_labelled.tif]

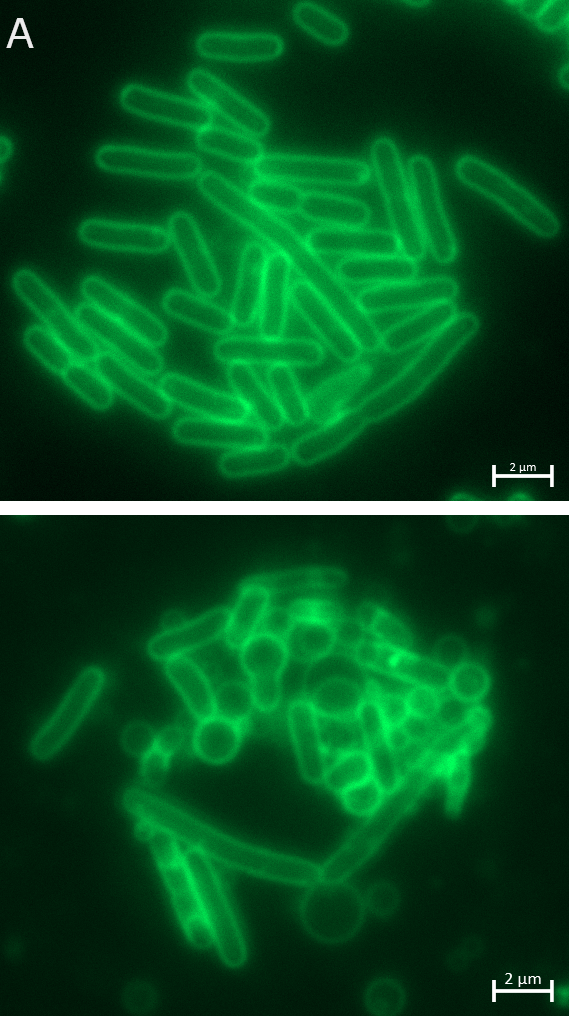

Supplement: Supplementary file 5 — Source data Fig. 4 [file 44320_2024_35_MOESM5_ESM.zip › MSB-2024-12200_SourceDataFor_Fig4A_labelled.tif]

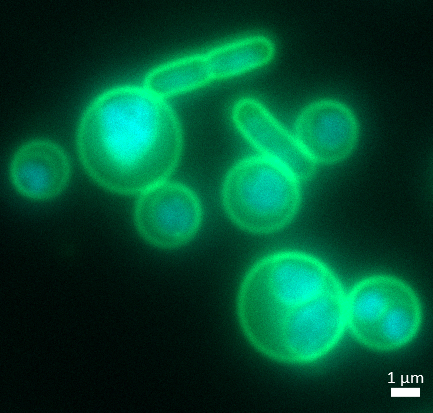

Supplement: Supplementary file 6 — EV Figure Source Data [file 44320_2024_35_MOESM6_ESM.zip › MSB-2024-12200_SourceDataFor_EV4C_allchannels.tif]

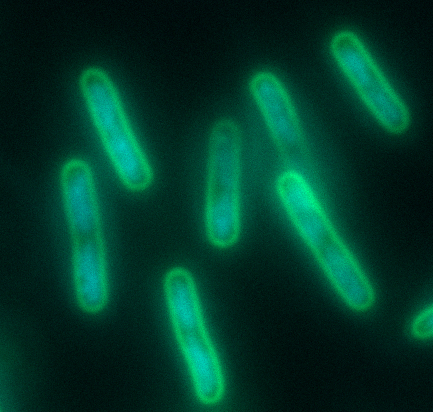

Supplement: Supplementary file 6 — EV Figure Source Data [file 44320_2024_35_MOESM6_ESM.zip › MSB-2024-12200_SourceDataFor_EV4C_top_allchannels.tif]

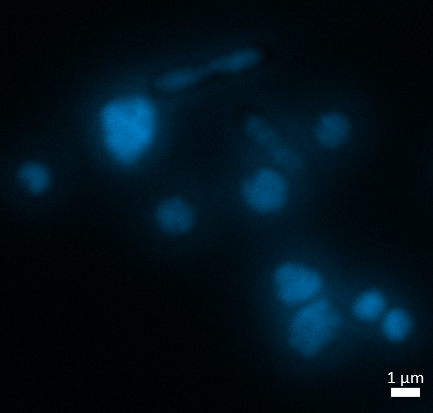

Supplement: Supplementary file 6 — EV Figure Source Data [file 44320_2024_35_MOESM6_ESM.zip › MSB-2024-12200_SourceDataFor_EV4C_DAPI.tif]

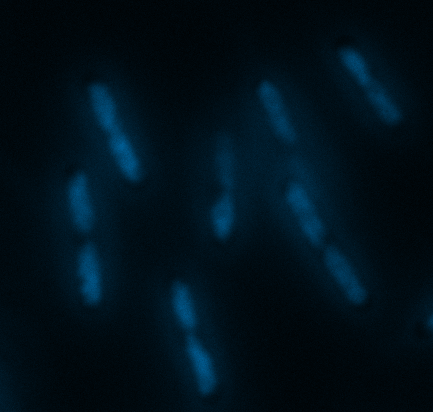

Supplement: Supplementary file 6 — EV Figure Source Data [file 44320_2024_35_MOESM6_ESM.zip › MSB-2024-12200_SourceDataFor_EV4C_top_DAPI.tif]
