# Supplementary material for: Identification of type VI secretion system effector-immunity pairs using structural bioinformatics
Source: Mol Syst Biol. 2024 Apr 24;20(6):6. doi: 10.1038/s44320-024-00035-8 (PMC11148199; doi:10.1038/s44320-024-00035-8)
Supplement: Supplementary file 8 — Expanded View Figures [file 44320_2024_35_MOESM8_ESM.pdf]

Expanded View Figures

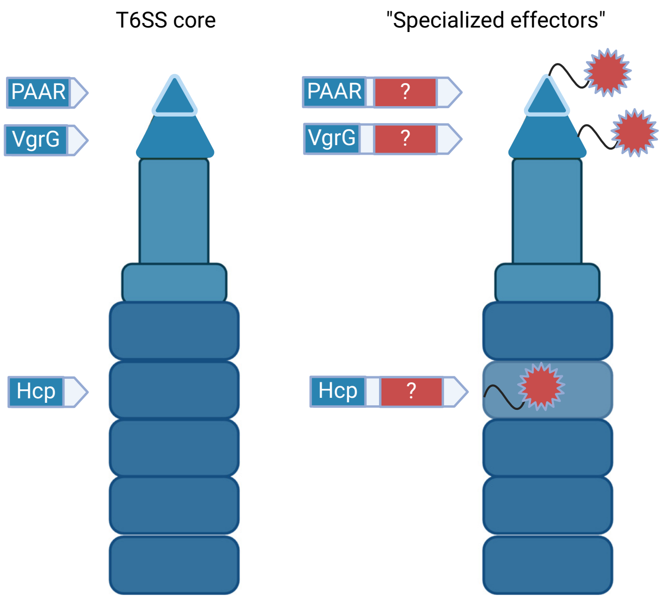

Figure EV1. T6SS structure and specialized effectors.

The T6SS is a contractile injection system made up of multiple components, including a donut-shaped tube component from hexamers of Hcp, and a sharp tip component made of VgrG and PAAR; we call these core T6SS components (right). Sometimes, these components have long C-terminal extensions that encode protein domains with T6SS effectors (left, red). Structurally, this results in what are called "specialized effector" proteins (sometimes referred to as "evolved" effectors). The C-terminal extensions lead to the loading of the C-terminal effector domain onto the T6SS, simply due to the fact that it is covalently attached to N-terminal core T6SS domains. The genetic organization of an N-terminal core domain and a C-terminal effector domain provides a characteristic genetic signature making specialized T6Es easily identifiable in large genomic databases.

 © The Author(s)

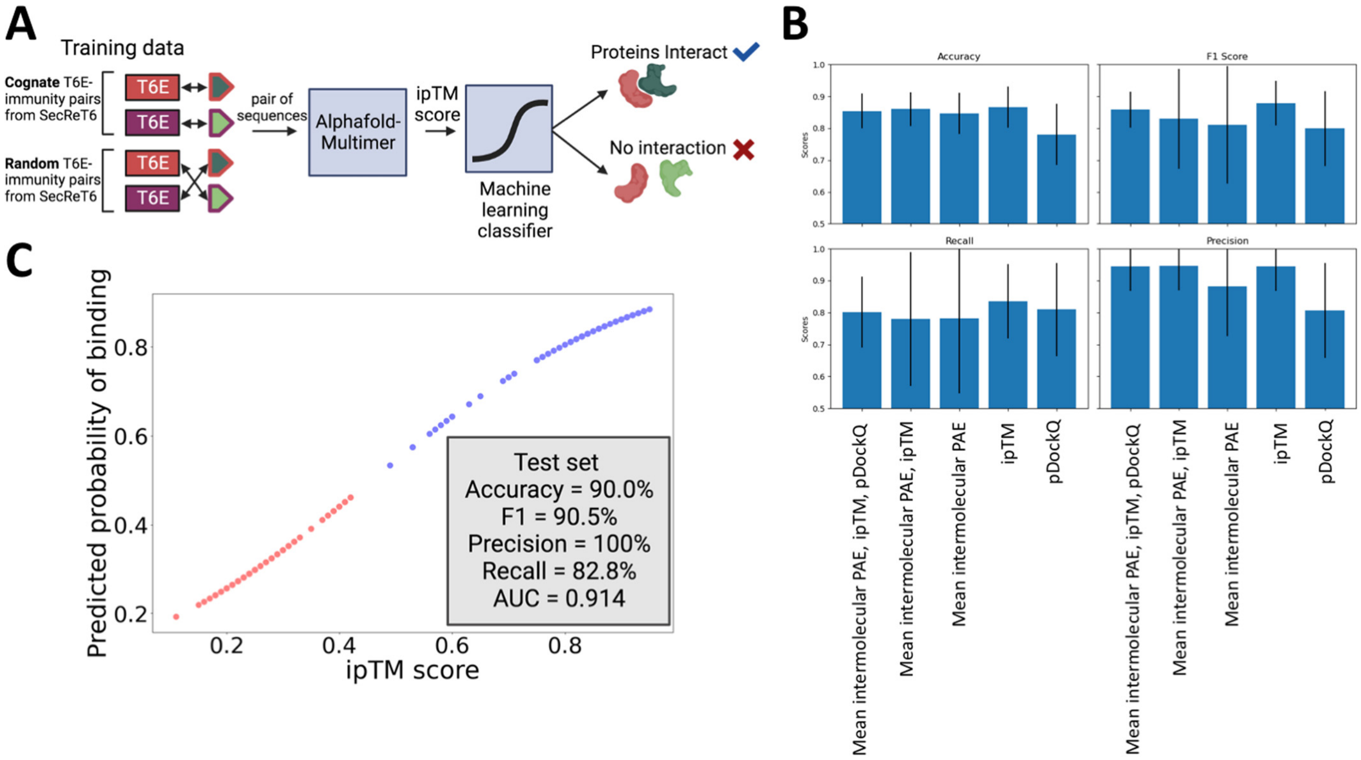

Figure EV2. Training a logistic regression model for classification of T6E-immunity interaction.

(**A**) Pipeline for training logistic regression model. T6E-immunity pairs from the SecReT6 database (Zhang et al, 2023; Li et al, 2015) were used as positive set input to Alphafold-Multimer, while shuffled (randomized) pairs of noncognate T6Es and immunity proteins were used as negative set inputs. The model was trained on ipTM score, a default output of Alphafold-Multimer. (**B**) Various iterations of the model were built, including based on multiple inputs beyond ipTM, like Mean intermolecular PAE, and pDockQ, and combinations of these inputs. ipTM alone was chosen in the end because it alone equaled or outperformed the other inputs in terms of accuracy, precision, F1 score, and recall. Bars represent mean, error bars represent standard deviation of $k = 10$ instances of k-fold validation performed on a dataset of $n = 164$ pairs (95 in positive set, 69 in the negative set). (**C**) Trained model evaluated on both training and test set reveals the shape of the trained logistic regression curve. Blue dots are predicted protein interactions, and red are predicted non-interactions. Inset shows test set accuracy, F1, precision, recall, and AUC values.

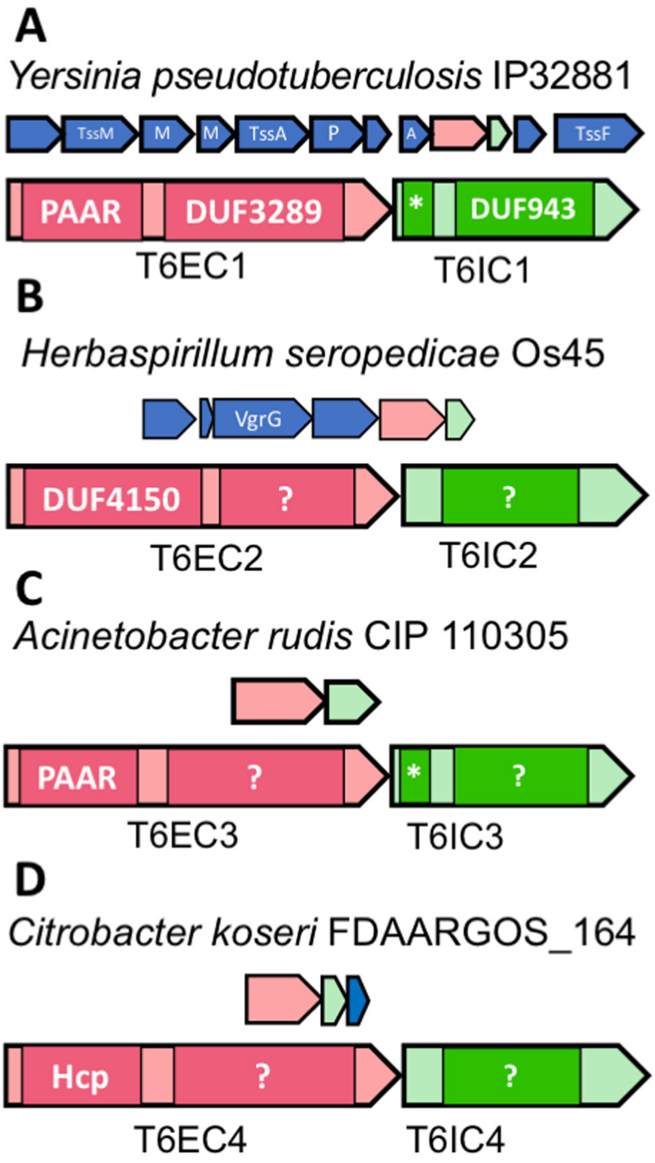

Figure EV3. T6EC1-4 and T6IC1-4 are putative T6E-immunity pairs.

Genes in operons are shown on top, and protein/domain architecture is shown on the bottom of each panel. (**A**) T6EC1 and T6IC1 are encoded in a T6SS operon (top; M = TssM, P = PAAR, A = TssA). T6IC1 has an N-terminal transmembrane domain signal, marked with an asterisk. The transmembrane prediction is as follows: residues 1–6 are inside, residues 7–26 are transmembrane helices, and residues 27–157 are outside (i.e., in the periplasm). (**B**) T6EC2 and T6IC2 are in an orphan/auxiliary operon containing a VgrG gene. (**C**) T6IC3 has an N-terminal signal sequence strongly suggesting it is localized to the periplasm. (**D**) T6EC4 and T6IC4 are in an orphan/auxiliary operon.

 © The Author(s)

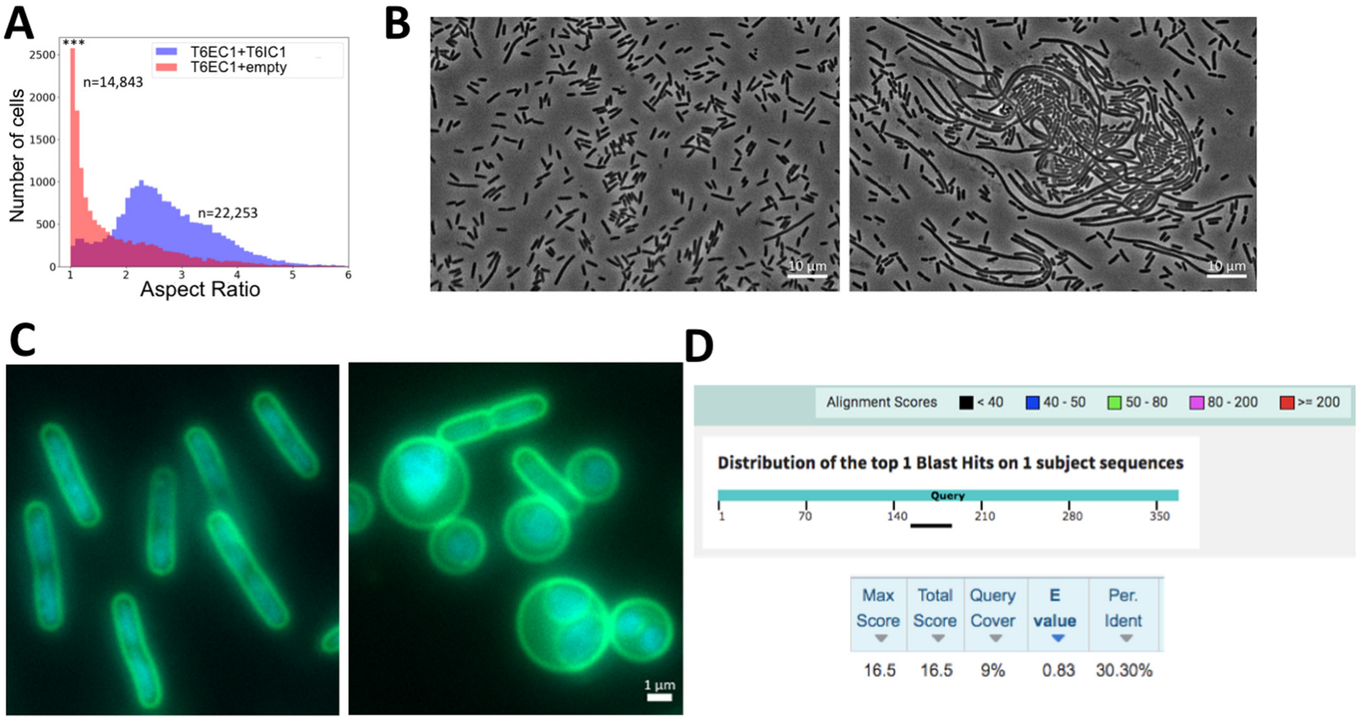

Figure EV4. Experimental exploration of T6EC functions.

(**A**) Histogram of aspect ratio (a measure of length/width). Blue = both T6EC1 and T6IC1, Red = T6EC1 only, purple = overlap of histograms. An aspect ratio of 1 is a circle (equal length and width). Asterisks indicate statistical significance (Mann–Whitney $U = 258904636$, $P = 1E\text{-}1871$). (**B**) Expression of T6EC2 results in filamentation. *E. coli* with an empty pBAD24 and pET29b vectors (left) or pBAD24 with T6EC2 and an empty pET29b vector (right) was imaged after 1 h of incubation with IPTG and arabinose. (**C**) *E. coli* BL21 expressing pBAD24 and pET29b empty vectors (left) or pBAD24 with T6EC3 and pET29b with T6IC3 after 15 min of induction with 0.01 mM IPTG and 0.2% Arabinose (right). Note the immunity protein is expressed but does not cause full saving activity. The same cells are shown in Fig. 4B, but here, the signal is an overlay of membrane and DNA signals from FM1-43 and DAPI, respectively. (**D**) Sequence-sequence search by BLAST of T6EC4 and TseT from *Pseudomonas aeruginosa* PAO1 has no significant similarity. Source data are available online for this figure.
